# Comparative Chloroplast Genomics of Dipsacales Species: Insights Into Sequence Variation, Adaptive Evolution, and Phylogenetic Relationships

**DOI:** 10.3389/fpls.2018.00689

**Published:** 2018-05-23

**Authors:** Wei-Bing Fan, Ying Wu, Jiao Yang, Khurram Shahzad, Zhong-Hu Li

**Affiliations:** Key Laboratory of Resource Biology and Biotechnology in Western China, Ministry of Education, College of Life Sciences, Northwest University, Xi’an, China

**Keywords:** Adoxaceae, Caprifoliaceae, chloroplast genome, Dipsacales, phylogenetic relationship, positive selection

## Abstract

In general, the chloroplast genomes of angiosperms are considered to be highly conserved and affected little by adaptive evolution. In this study, we tested this hypothesis based on sequence differentiation and adaptive variation in the plastid genomes in the order Dipsacales. We sequenced the plastid genomes of one Adoxaceae species and six Caprifoliaceae species, and together with seven previously released Dipsacales chloroplasts, we determined the sequence variations, evolutionary divergence of the plastid genomes, and phylogeny of Dipsacales species. The chloroplast genomes of Adoxaceae species ranged in size from 157,074 bp (*Sinadoxa corydalifolia*) to 158,305 bp (*Sambucus williamsii*), and the plastid genomes of Caprifoliaceae varied from 154,732 bp (*Lonicera fragrantissima* var. *lancifolia*) to 156,874 bp (*Weigela florida*). The differences in the number of genes in Caprifoliaceae and Adoxaceae species were largely due to the expansion and contraction of inverted repeat regions. In addition, we found that the number of dispersed repeats (Adoxaceae = 37; Caprifoliaceae = 384) was much higher than that of tandem repeats (Adoxaceae = 34; Caprifoliaceae = 291) in Dipsacales species. Interestingly, we determined 19 genes with positive selection sites, including three genes encoding ATP protein subunits (*atpA*, *atpB*, and *atpI*), four genes for ribosome protein small subunits (*rps3*, *rps7*, *rps14*, and *rps15*), four genes for photosystem protein subunits (*psaA*, *psaJ*, *psbC*, and *pabK*), two genes for ribosome protein large subunits (*rpl22* and *rpl32*), and the *clpP*, *infA*, *matK*, *rbcL*, *ycf1*, and *ycf2* genes. These gene regions may have played key roles in the adaptation of Dipsacales to diverse environments. In addition, phylogenetic analysis based on the plastid genomes strongly supported the division of 14 Dipsacales species into two previously recognized sections. The diversification of Adoxaceae and Caprifoliaceae was dated to the late Cretaceous and Tertiary periods. The availability of these chloroplast genomes provides useful genetic information for studying taxonomy, phylogeny, and species evolution in Dipsacales.

## Introduction

Traditionally, the order Dipsacales comprises the families Valerianaceae, Dipsacaceae, Adoxaceae, and Caprifoliaceae *sensu lato* (including Linnaeaceae, Diervillaceae, and Caprifoliaceae *sensu stricto*) (Cronquist, 1979; Thorne, 1992). The evolutionary relationships among some Dipsacales species have been revised based on studies of their taxonomy and phylogeny. For example, the families Linnaeaceae, Diervillaceae, and Caprifoliaceae *s. s.* have been renamed as Linnaeeae, Diervilleae, and Caprifolieae, respectively, and they constitute the new family Caprifoliaceae *s. l.* (Donoghue et al., 1992; Olmstead et al., 2000; Bell et al., 2001; Zhang et al., 2003). The general consensus is that Dipsacales comprises a monophyletic taxon with two major lineages (Donoghue et al., 1992; Olmstead et al., 1993, 2000; Zhang et al., 2003): (i) the large clade Caprifoliaceae containing Diervilleae, Caprifolieae, Linnaeeae, Morinaceae, Valerianaceae, and Dipsacaceae; and (ii) the smaller clade comprising the family Adoxaceae, which contains the genera *Viburnum*, and *Sambucus*, *Sinadoxa*, *Tetradoa*, and *Adoxa*. Within Adoxaceae, analyses of several data sets (e.g., morphological evidence as well as internal transcribed spacer, mitochondrial, and chloroplast (cp) DNA sequences) have suggested that the genera *Sambucus* and *Viburnum* (belonging to the traditional Caprifoliaceae) have a close relationship (Eriksson and Donoghue, 1997; Donoghue et al., 2001; Zhang et al., 2002; Winkworth et al., 2008). Within Caprifoliaceae, the genera *Lonicera* and *Triosteum* have a very close relationship (Donoghue et al., 2001; Zhang et al., 2002; Winkworth et al., 2008). In addition, other studies based on cp DNA regions *trnL-trnF* and *ndhF*, as well as mitochondrial DNA sequence variations have suggested that the genera *Dipelta* and *Kolkwitzia* have a very close evolutionary relationship (Donoghue et al., 2001; Zhang et al., 2002; Winkworth et al., 2008). These previous studies have resolved the basic phylogenetic relationships among Dipsacales, but the interspecific divergence and lineage structure of some main genera (e.g., *Dipelta*, *Kolkwitzia*, and *Weigela*) still remain largely controversial.

Many studies have focused on studying the origin and divergence history of Dipsacales as an important angiosperm clade. For example, Backlund (1996) employed DNA sequences and several important fossil points to estimate the origin of Dipsacales species as around 60–70 million years ago (mya) during the late Cretaceous or early Tertiary. Wikström et al. (2001) used the non-parametric rate smoothing method to estimate the origin of Dipsacales as 85–90 mya. In addition, Bell and Donoghue (2005) used the relaxed assumption of rate constancy among lineages to estimate the ages of major lineages and suggested a late Cretaceous origin for Dipsacales. Studies of the divergence times for Dipsacales based on the DNA sequence datasets have progressed greatly, but the divergence dates of the major lineages within Dipsacales remain unclear.

Chloroplasts are multifunctional organelles in plant cells with critical roles in photosynthesis and carbon fixation (Wicke et al., 2011; Daniell et al., 2016). Chloroplasts possess their own genetic material, which exists as a quadripartite circular molecule of double-stranded DNA containing two copies of inverted repeats (IRs) separated by two regions: the large (LSC) and small (SSC) single copy regions (Huotari and Korpelainen, 2012). Most angiosperm cp genomes are remarkably conserved in terms of their structure, gene content, and order (Wicke et al., 2011). The cp genomes of land plants usually encode 110–130 genes with sizes in the range of 120–160 kb (Zhang et al., 2014). In general, plant cp genomes are recombination-free, maternally inherited, and with low rates of nucleotide substitutions, which make them valuable sources of genetic markers for phylogenetic and population genetic analyses (Korpelainen, 2004; Ravi et al., 2008). In recent years, cp genomes have been used widely to study phylogenetic relationships and species evolution in different taxa, as well as for constructing the phylogenetic lineages of angiosperms. For example, studies based on cp genome datasets have shown that Chloranthaceae and Magnoliids are sisters to a clade of monocots, as well as eudicots including Ceratophyllaceae (Moore et al., 2007). In addition, orchids and grasses together form a monophyletic group nested within the remaining angiosperms (Chang et al., 2006). Similarly, important advances have been made based on the complete cp genomes in elucidating the relationships within the larger monocot (Graham et al., 2006) and asterid (Bremer et al., 2002) clades. Recently, several cp genome sequences have been published for Dipsacales species (Wang Y. et al., 2016; Bai et al., 2017; He et al., 2017). However, there has been little research into the phylogenetic evolution and interspecific divergence of Dipsacales species due to the lack of conjoint analyses of large cp genome datasets.

In general, the chloroplast genomes of angiosperms have slow substitution rates and they are affected little by adaptive evolution (Erixon and Oxelman, 2008). Excluding genes that have evolved very rapidly, non-synonymous nucleotide substitutions have occurred less frequently than synonymous substitutions due to the action of purifying selection (Ivanova et al., 2017). Previous studies have suggested that plant cp genes have lower rates of synonymous nucleotide substitutions than nuclear genes (Palmer, 1985; Wolfe et al., 1987). In addition, positive selection is expected to speed up non-synonymous substitution rates whereas synonymous rates are expected to be unaffected. However, until recently, adaptive evolution by positive selection had rarely been determined in Dipsacales cp genes.

In the present study, we investigated adaptive evolution in the cp genomes of Dipsacales. We collected materials from one Adoxaceae species and six Caprifoliaceae species, and we assembled and annotated their complete cp genomes, before comparing these cp genomes and other published cp genomes to explore genome differentiation and sequence divergence in Dipsacales. We also identified the variant hotspot regions in cp genomes and reconstructed the phylogenetic relationships and molecular divergence dates for the major lineages within the order Dipsacales.

## Materials and Methods

### Sample Collection, Genome Sequencing, and Assembly

Fresh leaves from one Adoxaceae species, *Viburnum betulifolium*, and six Caprifoliaceae species, *Lonicera fragrantissima* var. *lancifolia*, *Lonicera stephanocarpa*, *Lonicera tragophylla*, *Triosteum pinnatifidum*, *Weigela florida*, and *Dipelta floribunda*, were collected in Shaanxi Province in 2016 (**Supplementary Table S1**). Voucher specimens of each sample were deposited in the Key Laboratory of Resource Biology and Biotechnology in Western China (Xi’an, China). Total genomic DNA was isolated from 1 g of each fresh leaf sample using the modified CTAB method (Doyle, 1987). In addition, we downloaded the available complete cp genomes of seven other Dipsacales species from GenBank (*Viburnum utile*, NC_032296; *Sambucus williamsii*, NC_033878; *Sinadoxa corydalifolia*, NC_032040; *Lonicera japonica*, NC_026839; *Kolkwitzia amabilis*, NC_029874; *Adoxa moschatellina*, KX258652; and *Trachelium caeruleum*, NC_010442). The cp genomes of *Helianthus annuus* (NC_007977) and *Guizotia abyssinica* (NC_010601) were also downloaded for subsequent analyses.

After extracting the genomic DNA, approximately 5–10 μg of DNA was sheared, before adapter ligation and library amplification. The fragmented DNA was subjected to library preparation and paired-end read (PE150/PE125) sequencing was then conducted with the Illumina Hiseq 2500 platform. Raw reads were filtered to remove sequences shorter than 50 bp and adapter sequences, using the NGSQCToolkit_v2.3.3 tool (Patel and Jain, 2012). The Dipsacales cp genomes were then reconstructed by *de novo* assembly combined with reference-based assembly. We aligned the short reads obtained from the Illumina sequencing to the reference chloroplast genomes (*L. japonica*, *K. amabilis*, and *V. utile*) using Bowtie 2.2.6 (Langmead and Salzberg, 2012). Then, the cp reads that mapped to the reference genome were extracted to be used as input for *de novo* assembly using SPAdes 3.9.0 (Bankevich et al., 2012). For the reference-based assembly, the clean reads for *L. fragrantissima* var. *lancifolia*, *L. stephanocarpa*, *L. tragophylla*, *T. pinnatifidum*, *W. florida*, *D. floribunda*, and *V. betulifolium* were first assembled using MIRA 4.0.2 (Chevreux et al., 2004), where the references comprised the cp genomes of the closely related species *Lonicera japonica* (NC_026839), *Kolkwitzia amabilis* (NC_029874), and *Viburnum utile* (NC_032296). Subsequently, some ambiguous regions were selected for extension by using a baiting and iteration method with the MITObim v1.8 program (Hahn et al., 2013). The contigs obtained were used to generate consensus sequences with Geneious R v9.0.5 (Kearse et al., 2012). A small number of gaps and low coverage regions in the assembled cp genomes were validated using the Sanger sequencing method, with primers (**Supplementary Table S2**) developed using Primer3 (Untergasser et al., 2012).

### Genome Annotation

The consensus sequences were imported into the online program Dual Organellar Genome Annotator (DOGMA, Wyman et al., 2004) for gene annotation, guided by the other cp genomes. In addition, all of the tRNA genes were further verified using tRNAscan-SE1.21 (Schattner et al., 2005). Sequences were aligned using the Mauve program to compare the structure and gene contents within the genomes (Darling et al., 2004). We also re-annotated the sequences downloaded from NCBI Genbank before using them in our analyses. The newly obtained chloroplast genomes and the raw reads of Dipsacales species were submitted into the GenBank under accession numbers are MG738664-MG738664 and SRR6898410-SRR6898416, respectively. Finally, circular plastid genome maps were drawn using OGDRAW (Lohse et al., 2013).

### Repeat Element Analysis

Repeat motifs are very useful markers with important roles in phylogenetic analysis (Cavalier-Smith, 2002; Nie et al., 2012). In general, large repeated elements comprise dispersed, palindromic, and tandem repeats. Tandem repeat in DNA is the pattern of two or more adjacent, approximate copies of nucleotides. Dispersed repeats are nucleotide sequences present in multiple copies in the genome. Palindromic repeat is an inverted repeat sequence with no intervening nucleotides between the initial sequence and its downstream reverse complements. In order to identify repeat elements, the web-based REPuter program (Kurtz et al., 2001) was used to analyze the dispersed and palindromic repeats based on the following conditions; (1) Hamming distance = 1; (2) sequence identity ≥ 90%; and (3) minimum repeat size = 30 bp. In addition, the tandem repeat sequences (>10 bp in length) were detected using the online Tandem Repeats Finder program (Benson, 1999), where the alignment match, mismatch, and indel parameters were set as two, seven, and seven, respectively. The minimum alignment score and maximum period size were 80 and 500, respectively.

### Sequence Divergence Analysis

Alignments of the 14 Dipsacales complete cp genome sequences were visualized using mVISTA (Frazer et al., 2004). We extracted all the coding regions and intergenic spacers to examine regions of divergence within Adoxaceae and Caprifoliaceae for further phylogenetic analysis. The percentage of variable sites was calculated within each homologous region.

### Adaptive Evolution Analysis

To analyze the non-synonymous (dN) and synonymous (dS) substitution rates, and their ratio (ω = dN/dS), the same unique functional protein coding sequences for each gene were extracted and aligned separately using Geneious R v9.0.5, and maximum likelihood phylogenetic trees were reconstructed based on the complete cp genomes using RAxML v 7.2.8 (Stamatakis, 2006). The values of dN, dS, and ω for each protein-coding exon were calculated using the site-specific model implemented in the codeml package (seqtype = 1, model = 0, NSsites = 1, 2, 7, 8) in PAML4.7 (Yang et al., 2005). This model allowed the ω ratio to vary among sites with a fixed ω ratio in all branches in order to test for site-specific evolution in the gene phylogeny (Yang and Nielsen, 2002). Two likelihood ratio tests were performed to check for the presence of positively selected sites: M1 (neutral) vs. M2 (positive selection), and M7 (beta) vs. M8 (beta and ω), which were compared using site-specific models (Yang and Nielsen, 2002; Yang et al., 2005). Model M1 distinguished two site classes with ω < 1 and ω = 1, and model M2 allowed for a third site class with ω > 1. Models M7 and M8 both described the distribution of ω as a beta function. The beta null model M7 restricted ω to (0, 1), and the alternative beta and ω model M8 allowed for positively selected extra site classes. Only candidate sites for positive selection with significant support from the posterior probability [*p*_(ω__>__1)_ ≥ 0.99]; Bayes Empirical Bayes approach) identified by M2 and M8 were considered further.

### Phylogenetic Analysis

We used the 16 cp genomes to analyze the phylogenetic relationships among Dipsacales species, including six Adoxaceae species, eight Caprifoliaceae species, and two outgroups *Guizotia abyssinica* (NC_010601) and *Helianthus annuus* (NC_007977). The phylogenetic analysis were conducted based on the following five data partitions: (1) complete cp genomes; (2) protein-coding sequences; (3) LSC region; (4) IR region; and (5) SSC region. These regions were aligned using Mauve (Darling et al., 2004) and the best-fitting model was determined using MrModeltest 2.3 (Nylander, 2004). Maximum likelihood analysis was conducted using the program RAxML v 7.2.8 (Stamatakis, 2006) with 1,000 bootstrap replicates. Bayesian inference was performed using MrBayes v3.1.2 (Ronquist and Huelsenbeck, 2003) with the following settings: Markov chain Monte Carlo simulations for 1,000,000 generations with four incrementally heated chains, starting from random trees and sampling one out of every 1,000 generations. The first 25% of the trees were regarded as burn-ins (Meng et al., 2008; Ma et al., 2014).

### Divergence Time Estimation

The divergence times between lineages were estimated using a Yule process speciation prior and the uncorrelated lognormal model of rate change with a relaxed clock in BEAST v1.8.0 (Drummond et al., 2012). We set the stem of *Dipelta* with: lognormal mean = 0, *SD* = 1.0, offset = 36 mya; and the Dipsacales node was constrained to: 79.9 mya, with a normal prior, mean = 79.9 mya, *SD* = 5. The GTRAGMMA nucleotide substitution model was selected using MrModeltest 2.3 (Nylander, 2004). A normal prior probability distribution was used to consider the uncertainty of prior knowledge. The analyses were run for 20,000,000 generations and the parameters were sampled every 5,000 generations. The effective sample size (>200) was determined using Tracer v 1.6 (Drummond et al., 2012) and the first 10% of the samples were discarded as burn-ins. Tree Annotator v.1.8.0 (Drummond et al., 2012) was used to summarize the set of post burn-in trees and their parameters in order to produce a maximum clade credibility chronogram showing the mean divergence time estimates with 95% highest posterior density (PHD) intervals. FigTree V1.3.1 (Drummond et al., 2012) was used for visualize the resulting divergence times.

## Results

### Features of cp Genomes of 14 Dipsacales Species

**FIGURE 1:**
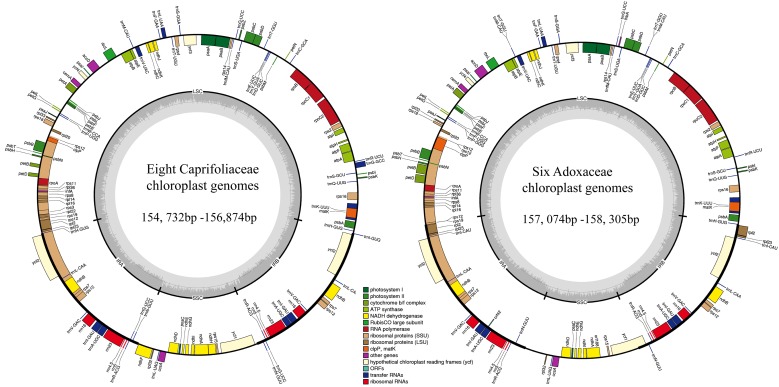
Chloroplast genome map for eight Caprifoliaceae species and six Adoxaceae species. Genes located outside the outer rim are transcribed in a counterclockwise direction, whereas genes inside the outer rim are transcribed in a clockwise direction. The colored bars indicate known different functional groups. The dashed gray area in the inner circle shows the percentage GC contents of the corresponding genes. LSC, SSC, and IR denote large single copy, small single copy, and inverted repeat, respectively.

In this study, we determined the structural characteristics and gene contents of the complete cp genomes of six Adoxaceae species (*V. betulifolium*, MG738665; *V. utile*, *S. williamsii*, *S. corydalifolia*, *A. moschatellina*, and *T. omeiensis*) and eight Caprifoliaceae species (*L. fragrantissima* var. *lancifolia*, MG738669; *L. stephanocarpa*, MG738668; *L. tragophylla*, MG738667; *T. pinnatifidum*, MG738666; *W. florida*, MG738664; *D. floribunda*, MG738670; *L. japonica*, and *K. amabilis*) within the order Dipsacales. The cp genomes of the six Adoxaceae species ranged in size from 157,074 bp (*S. corydalifolia*) to 158,305 bp (*S. williamsii*), and the eight Caprifoliaceae cp genomes ranged from 154,732 bp (*L. fragrantissima* var. *lancifolia*) to 156,874 bp (*W. florida*) (**Figure 1** and **Table 1**). All of the cp genomes had a typical quadripartite structure and they were similar to those of most land plants. The LSC length in the six Adoxaceae cp genomes ranged from 86,171 bp (*S. corydalifolia*) to 86,810 bp (*S. williamsii*), and the SSC and IR lengths ranged from 18,338 bp (*V. betulifolium*) to 18,993 bp (*S. williamsii*) and 26,112 bp (*A. moschatellina*) to 26,462 bp (*V. betulifolium*), respectively. All eight Caprifoliaceae cp genomes had an LSC region of 88,504 bp (*L. fragrantissima* var. *lancifolia*) to 89,964 bp (*K. amabilis*), an SSC region of 18,672 bp (*L. japonica* to 20,543 bp (*T. pinnatifidum*), and an IR of 22,673 bp (*T. pinnatifidum*) to 23,946 bp (*K. amabilis*) (**Table 1**). In addition, the six Adoxaceae cp genomes encoded 129 functional genes, with 84 protein-coding genes, 37 tRNA genes, and eight ribosomal RNA genes. The eight Caprifoliaceae cp genomes encoded 128 genes (**Supplementary Table S8**), with 82 protein-coding genes, 37 tRNA genes, eight ribosomal RNA genes and one pseudogene. The cp genomes of 14 Dipsacales species had the same average GC contents (mean = 38.23%). The GC contents of the SC regions in the six Adoxaceae (mean = 33.9%) and eight Caprifoliaceae (mean = 34.96%) species were lower than those of the IR regions (mean = 43.9%, 43.7%, respectively) (**Table 1**). The high GC percentage in the IR regions was possibly due to the presence of four rRNA genes in these regions. These results are similar to a previous report of a high GC percentage in the IR regions (Qian et al., 2013).

**Table 1:** Features of chloroplast genomes in six Adoxaceae and eight Caprifoliaceae species.

Species	Size (bp)	LSC (bp)	SSC (bp)	IR (bp)	Number of total genes	Number of PCGs	Number of tRNA genes	Number of rRNA genes	Overall GC content (%)	GC content of LSC (%)	GC content of SSC (%)	GC content of IR (%)
*Sinadoxa corydalifolia*	157,074	86,171	18,671	26,247	129	84 (6)	37 (7)	8 (4)	37.7	35.9	31.4	42.8
*Adoxa moschatellina*	157,238	86,341	18,674	26,112	129	84 (6)	37 (7)	8 (4)	37.8	36.0	31.5	42.9
*Tetradoxa omeiensis*	157,502	86,527	18,682	26,147	129	84 (6)	37 (7)	8 (4)	37.7	35.9	31.4	42.8
*Sambucus williamsii*	158,305	86,810	18,993	26,281	129	84 (6)	37 (7)	8 (4)	37.9	36.2	31.7	43.0
*Viburnum utile*	157,620	86,579	18,795	26,123	129	84 (6)	37 (7)	8 (4)	37.9	36.4	32.0	43.2
*Viburnum betulifolium*	158,023	86,761	18,338	26,462	129	84 (6)	37 (7)	8 (4)	38.1	36.4	32.1	42.9
*Lonicera fragrantissima* var. *lancifolia*	154,732	88,504	18,766	23,731	128	82 (4)	37 (7)	8 (4)	38.3	36.8	32.9	43.3
*Lonicera stephanocarpa*	155,056	88,912	18,763	23,690	128	82 (4)	37 (7)	8 (4)	38.3	36.8	32.8	43.4
*Lonicera tragophylla*	155,545	89,299	18,728	23,759	128	82 (4)	37 (7)	8 (4)	38.5	37.0	33.1	43.5
*Lonicera japonica*	155,078	88,858	18,672	23,774	128	82 (4)	37 (7)	8 (4)	38.6	37.1	33.4	43.5
*Kolkwitzia amabilis*	156,702	89,964	18,846	23,946	128	82 (4)	37 (7)	8 (4)	38.4	36.5	33.2	44.1
*Triosteum pinnatifidum*	154,896	89,007	20,543	22,673	128	82 (4)	37 (7)	8 (4)	38.5	36.8	33.9	44.0
*Weigela florida*	156,874	89,763	20,073	23,019	128	82 (4)	37 (7)	8 (4)	38.1	36.4	33.1	43.6
*Dipelta floribunda*	155,370	89,431	19,052	23,378	128	82 (4)	37 (7)	8 (4)	38.4	36.4	33.1	44.2

*Numbers in brackets indicate the genes duplicated in the IR regions.*

### Repeat Element Analysis

**FIGURE 2:**
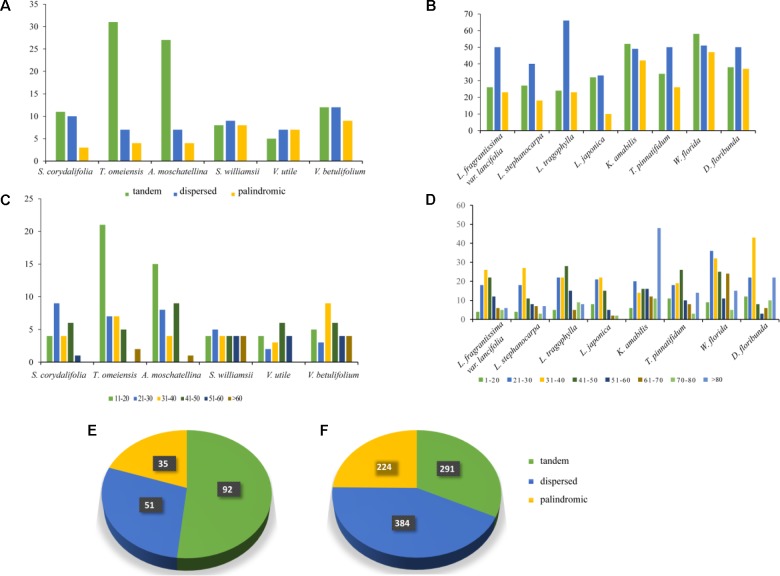
Maps obtained by repeat sequence analyses. **(A)** Histogram showing the number of repeats in the six Adoxaceae chloroplast genomes. **(B)** Histogram showing the number of repeats in the eight Caprifoliaceae chloroplast genomes. **(C)** Compositions of the repeats in six Adoxaceae species. **(D)** Compositions of the repeats in six Adoxaceae species. **(E)** Pie chart showing the numbers of the three repeat types in Adoxaceae. **(F)** Pie chart showing the numbers of the three repeat types in Caprifoliaceae.

We divided the repeats into three categories: tandem, dispersed, and palindromic (**Supplementary Tables S3**, **S4**). The number of tandem repeats (92) was higher than that of dispersed repeats (51) and palindromic repeats (35) in the six Adoxaceae species, and the number of dispersed repeats (384) was higher than that of tandem repeats (291) and palindromic repeats (224) in the eight Caprifoliaceae species (**Figure 2**). In addition, the total number of repeats in the six Adoxaceae species (178) was much lower than that in the eight Caprifoliaceae species (904). In the Adoxaceae family, the number of repeats was highest in *A. moschatellina* (42) and lowest in *V. utile* (19). In Caprifoliaceae, the numbers of all repeats, tandem repeats, and palindromic repeats were 155, 58, and 47 in *W. florida*, respectively, 141, 52, and 40 in *K. amabilis*, and 124, 38, and 37 in *D. floribunda*. The repeat units were mainly 21–50 bp regions in Caprifolieae and *W. florida*. In *K. amabilis*, most of the repeat units comprised 21–50 bp (48), followed by repeat units measuring 21–30 bp (20), and 0–20 bp (six). Within *D. floribunda*, most of the repeat units were 31–40 bp (43), followed by repeat units measuring > 80 bp (22), 21–30 bp (22), and 51–60 bp (three). Most of the repeats were distributed in intergenic or intron regions, and only a minority were located in gene regions in the order Dipsacales.

### Contraction and Expansion of IRs

**FIGURE 3:**
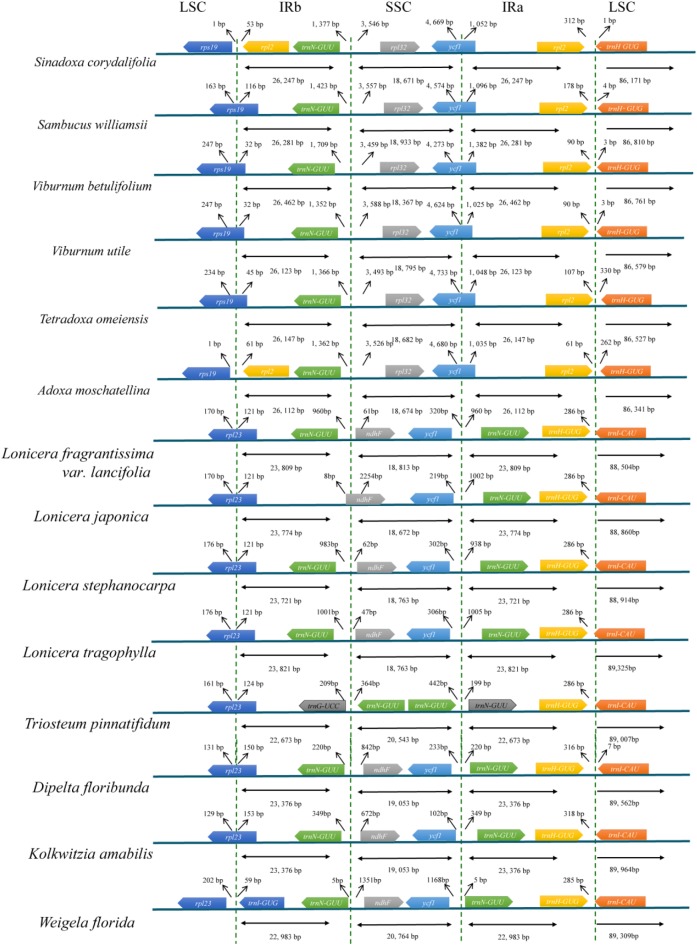
Comparison of the border positions of LSC, SSC, and IR regions in the chloroplast genomes in six Adoxaceae species and eight Caprifoliaceae species.

We analyzed the IR/single copy (SC) region border positions and their adjacent genes in the six Adoxaceae and eight Caprifoliaceae cp genomes (**Figure 3**). The *rpl2* and *rpl32* genes were detected around the junctions of the IRb/SSC and IRa/LSC regions in six Adoxaceae species, and the *ndhF* and *trnI-CAU* genes appeared in these two regions in eight Caprifoliaceae species. In addition, the IR/SC boundary structure was similar in six Adoxaceae species, where the *rps19* gene was located in the junction of the LSC/IRb region in *S. williamsii*, *V. betulifolium*, *V. utile*, and *T. omeiensis*, and the *rps19* gene was located in the LSC region in *S. corydalifolia* and *A. moschatellina*. There was high variability in the IRb/SSC and SSC/IRa boundaries in the eight Caprifoliaceae species. The junction position between IRb and SSC was located in the *ndhF* gene, except it was in the IRb region in *L. japonica*. In addition, the *trnN-GUU* gene extended into the SSC region and appeared twice in *T. pinnatifidum*.

### Sequence Divergence Analysis

**FIGURE 4:**
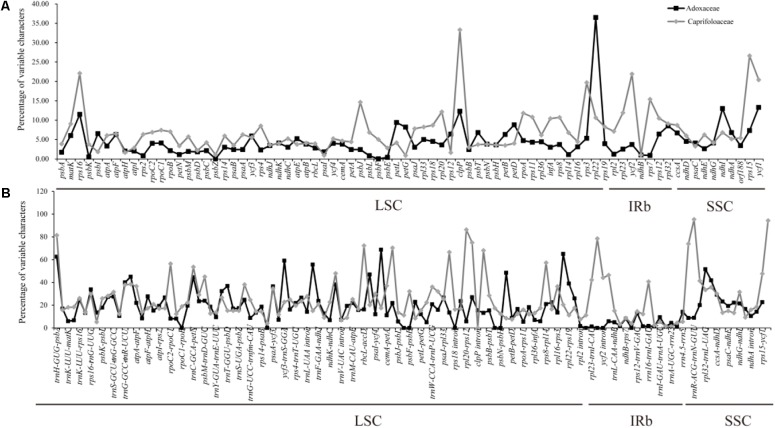
Percentages of variable characters in homologous regions among the chloroplast genomes of 14 Dipsacales species. **(A)** Coding region. **(B)** Non-coding region. The homologous regions are oriented according to their locations in the chloroplast genome.

The multiple complete cp genomes allowed us to estimate sequence variation. The divergence of sequences in the cp genomes of six Adoxaceae species and eight Caprifoliaceae species was plotted using the mVISTA program with annotations for *S. corydalifolia* as the reference (**Supplementary Figure S1**). In addition, the percentage variation was calculated in each of the 14 Dipsacales cp genomes (**Figure 4** and **Supplementary Table S5**). The results showed that the mean percentage of variation was 12.8% in the six Adoxaceae cp genomes and 18.77% in the eight Caprifoliaceae species. In addition, the percentage variations in the coding regions (Adoxaceae, mean = 4.56%; Caprifoliaceae, mean = 7.11%) were smaller than those in the non-coding regions (Adoxaceae, mean = 17.95%; Caprifoliaceae, mean = 24.72%). Interestingly, the single copy regions (mean = 12.75%) had a higher average percentage of variation than the inverted repeats (mean = 4.14%) in six Adoxaceae species. However, the variation in single copy regions (mean = 17.61%) was lower than that in inverted repeat regions (mean = 21.25%) in Caprifoliaceae. In the coding regions, the five genes with the greatest variability (>10%) were *rpl22*, *ndhI*, *ycf1*, *clpP*, and *rps16* in Adoxaceae, and the percentage variation in seven genes (*rps16*, *clpP*, *rps3*, *ycf2*, *rps7*, *rps15*, and *ycf1*) exceeded 15%. In non-coding regions, 11 regions (*trnH-GUG-psbA*, *trnR-UCU-atpA*, *trnC-GCA-petN*, *ycf3-trnS-GGA*, *trnL-UAA-trnF-GAA*, *accD-psaI*, *ycf4-cemA*, *psbH-petB*, *rpl32-trnL-UAG*, *trnL-UAG-ccsA*, and *rps3-rpl22*) in Adoxaceae and 16 regions (*trnH-GUG-psbA*, *rpoC2-rpoC1*, *trnC-GCA-petN*, *ndhC-trnV-UAC*, *rbcL-accD*, *accD-psaI*, *psbJ-psbL*, *rps18* intron, *infA-rps8*, *trnI-CAU-ycf2*, *ycf2-trnL-CAA*, *rrn5-trnR-ACG*, *trnR-ACG-trnN-GUU*, *trnN-GUU-rpl32*, *rps15-ycf1*, and *ycf1-trnN-GUU*) in Caprifoliaceae had high levels of variation (percentage variation > 40%, 45%, respectively).

### Positive Selection Analysis

We identified 19 genes with positively selected sites in the order Dipsacales (**Supplementary Tables S6**, **S7**). Interestingly, these genes included three ATP subunit genes (*atpA*, *atpB*, and *atpI*), four ribosome small subunit genes (*rps3*, *rps8*, *rps14*, and *rps15*), four photosystem subunit genes (*psaA*, *psaJ*, *psbC*, and *pabK*), two ribosome large subunit genes (*rpl22* and *rpl32*), and the *clpP*, *infA*, *matK*, and *rbcL* genes. In addition, according to the M2 and M8 models, the *ycf2* gene harbored 15 and 18 sites under positive selection, respectively, with five and 16 in *ycf1*, three and five in *rbcL*, one and three in *atpA*, and two and two in *clpP*, and the other 14 genes each had only one active site. Both likelihood ratio tests (M1 vs. M2; M7 vs. M8) supported the presence of positively selected codon sites (*p* < 0.01) (**Supplementary Table S9**).

### Phylogenetic Relationships

**FIGURE 5:**
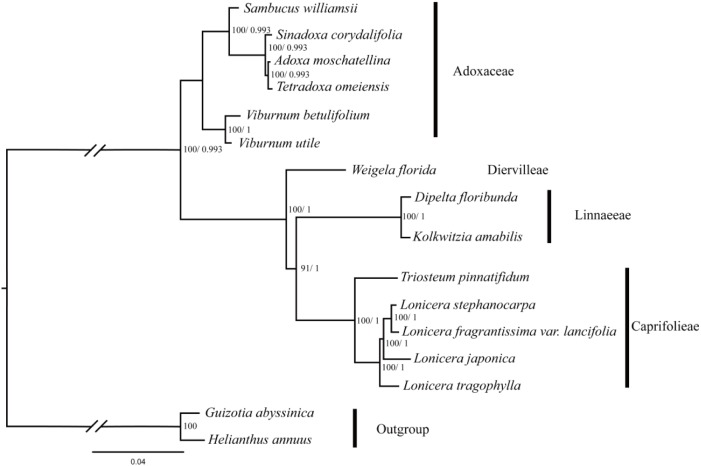
Phylogenetic tree obtained for 14 Dipsacales species based on the complete chloroplast genomes. The numbers to the left of the slashes on the braches show the bootstrap values obtained by maximum likelihood analyses, and those to the right show the posterior probabilities according to Bayesian inference.

In this study, five data partitions from the cp genomes of 16 Dipsacales species were used to reconstruct the phylogenetic relationships. The topologies obtained were largely consistent with the different datasets (**Figure 5** and **Supplementary Figure S2**) where two major clades were identified comprising a large clade and a small clade with 100% bootstrap values (except the LSC dataset which had a bootstrap value of 48%). A small clade in Adoxaceae included the genera *Viburnum*, *Sambucus*, *Adoxa*, *Tetradoxa*, and *Tetradoxa*. *V. utile* and *V. betulifolium* formed a clade with 100% bootstrap support. *Viburnum* and *Sambucus* were very closely related. In another clade, *D. floribunda* was placed as a sister to *K. amabilis* with high bootstrap values. *L. fragrantissima* var. *lancifolia* and *L. stephanocarpa* had close relationships with *L. japonica* and *L. tragophylla* in the genus *Lonicera*. In addition, *W. florida* was the earliest diverging lineage in the family Caprifolieae *s. s*.

### Divergence Time Estimation

**FIGURE 6:**
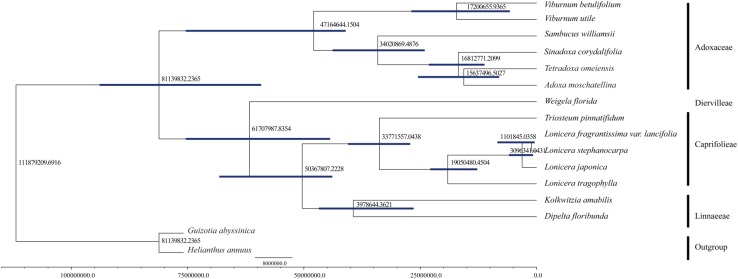
Molecular dating of 14 Dipsacales species based on the protein-coding sequences in chloroplast genomes.

We computed the molecular divergence dates of the 14 Dipsacales species based on the protein-coding sequences (**Figure 6**). The results showed that the divergence time between Adoxaceae and Caprifoliaceae was about 81.14 mya (95% PHD = 58.28–94.57 mya, calibration point = 79.9 mya), thereby suggesting a late Cretaceous origin for these two families within the order Dipsacales. The split of the *Viburnum* lineage to yield *Adoxa*, *Tetradoxa* and *Sinadoxa* occurred in the Eocene, with an estimated mean age of 47.16 mya (95% PHD = 42.08–76.45 mya). We estimated that *Sambucus* split from its ancestor about 34.02 mya (95% PHD = 24.36–44.13 mya). The diversification of *Sinadoxa*, *Tetradoxa*, and *Adoxa* occurred in the Miocene about 15–16 mya. Within Caprifoliaceae, the diversification of Diervilleae occurred in the Palaeocene, with an estimated mean age of 61.71 mya. Linnaeeae diversified from Caprifolieae in the Eocene (95% PHD = 25.75–46.99 mya, mean age of 39.79 mya). The divergence time of *L. tragophylla* (mean age of 19.05 mya) was much earlier than that of *L. japonica* (mean age = 3.10 mya), and that of *L. stephanocarpa* and *L. fragrantissima* var. *lancifolia* (mean age = 1.10 mya).

## Discussion

### Sequence Differentiation

In terms of genome size, the six Adoxaceae (mean = 157,755 bp) cp genomes were much larger than those of the eight Caprifoliaceae species (mean = 155,675 bp). Meanwhile, the sizes of inverted repeat regions in six Adoxaceae species (mean = 26,229 bp) were much larger than those of the eight Caprifoliaceae species (mean = 23,496 bp). The difference of these cp genomes size may have been due to the expansion and contraction of the border positions between the IR regions and SC regions (Wang and Messing, 2011). In general, most angiosperms contain 74 protein-coding genes, and evidence of gene loss-and-gain events and rearrangements is present in some species, although the cp genomes of land plants are considered highly conserved (Millen et al., 2001; Kim et al., 2009). In this study, we found that the Adoxaceae and Caprifoliaceae cp genomes encoded 84 and 82 protein-coding genes, respectively. In addition, the members of these two families all had 37 tRNA genes and eight rRNA genes. We detected only single copies of *rpl2* and *rpl23* in Caprifoliaceae, located at the LSC region, but two copies of these genes in Adoxaceae were located in the IR regions. Meanwhile, *rpl2* and *rpl23* was located in LSC/IR border positions, which possibly due to the expansion and contraction of the border regions (Wang and Messing, 2011). In addition, the *ndhF* gene only appeared in Caprifoliaceae and it was located around the junction of the IRb/SSC regions. *N. flexilis* and *P. stellaris* lacked the *ndh* gene, which may be related to their adaptation to a submerged environment (Peredo et al., 2013) and non-photosynthetic lifestyle (Straub et al., 2012). Similarly, *ndh* gene losses have been frequent in Orchidaceae (Kim et al., 2015), where Chang et al. (2006) found that the *ndhA*, *ndhF*, and *ndhH* genes had transferred to the nuclear genome in *Phalaenopsis aphrodite*. In addition, the *ndhF* gene plays a role in IR/SSC junction stability (Kim et al., 2015). We also found that the *accD* gene encoding a subunit of heteromeric cetyl-CoA carboxylase was present as a pseudogene in eight Caprifoliaceae species. Previous studies have also shown that the *accD* gene has been lost from some plant species (Downie and Palmer, 1992; Cosner et al., 1997), and that it is present as a pseudogene in *Jasminum nudiflorum*, *Trachelium caeruleum*, and *Eucommia ulmoides* (Wang L. et al., 2016).

### Divergence Hotspot Regions

In order to determine the divergence hotspots, we compared the whole cp genome sequences of the Adoxaceae and Caprifoliaceae species using mVISTA, where we computed the percentages of variable characters in coding and non-coding regions. Our results indicated that the proportions and distributions of the variable sites were strikingly different among the cp genomes. Overall, the proportion of variable sites was higher in the non-coding regions than the coding regions, which is generally consistent with most previous studies of the plastid genomes of angiosperms (Wolfe et al., 1987; Clegg et al., 1994; Perry and Wolfe, 2002; Jansen and Ruhlman, 2012; Huang et al., 2014). Interestingly, the SC regions (mean = 12.75%) had a higher average percentage of variation than the IRs (mean = 4.14%) in six Adoxaceae species. However, the variation in single copy regions (mean = 17.61%) was lower than that in inverted repeat regions (mean = 21.25%) in Caprifoliaceae, possibly due to the instability of the boundary of the IR region. Considering the proportion and number of variable sites, we propose 17 (*rpl22*, *ndhI*, *ycf1*, *trnH-GUG-psbA*, *trnR-UCU-atpA*, *trnC-GCA-petN*, *ycf3-trnS-GGA*, *trnL-UAA-trnF-GAA*, *accD-psaI*, *ycf4-cemA*, *psbH-petB*, *rpl32-trnL-UAG* and *trnL-UAG-ccsA*) and 23 (*rps16*, *clpP*, *rps3*, *ycf2*, *rps7*, *rps15*, *ycf1*, *trnH-GUG-psbA*, *rpoC2-rpoC1*, *trnC-GCA-petN*, *ndhC-trnV-UAC*, *rbcL-accD*, *accD-psaI*, *psbJ-psbL*, *rps18* intron, *infA-rps8*, *trnI-CAU-ycf2*, *ycf2-trnL-CAA*, *rrn5-trnR-ACG*, *trnR-ACG-trnN-GUU*, *trnN-GUU-rpl32*, *rps15-ycf1*, and *ycf1-trnN-GUU*) of the most variable hotspot regions as candidate DNA barcodes for Adoxaceae and Caprifoliaceae species for future studies. These regions may be very useful for assessing the phylogenetic relationships and interspecific divergence in Dipsacales species.

### Adaptive Selection

Synonymous and non-synonymous nucleotide substitution patterns are very important markers for gene evolution studies. In most genes, synonymous nucleotide substitutions have occurred more frequently than non-synonymous ones (Ogawa et al., 1999). Thus, a ratio of dN/dS < 1 indicates purifying selection, dN/dS *>* 1 denotes probable positive selection, and dN/dS values close to one indicate neutral evolution. Our analysis identified 19 genes with positive selection sites. These genes included three ATP subunit genes (*atpA*, *atpB*, and *atpI*), four ribosome small subunit genes (*rps3*, *rps7*, *rps14*, and *rps15*), four photosystem subunit genes (*psaA*, *psaJ*, *psbC*, and *pabK*), two ribosome large subunit genes (*rpl22* and *rpl32*), and the *clpP*, *infA*, *matK*, *rbcL*, *ycf1*, and *ycf2* genes. ATP synthase is essential during photosynthesis and it is usually the product of two genetic systems in plants (Westhoff et al., 1985). Six ATP subunit genes (*atpA*, *atpB*, *atpE*, *atpF*, *atpH*, and *atpI*) are encoded and synthesized in the chloroplasts (Westhoff et al., 1985), and three genes exhibited site-specific selection in this study. In addition, 21 genes were identified that encode ribosome subunits and four of these genes were under positive selection. Two photosystem I subunit genes (*psaJ* and *psaA*) and two photosystem II subunit genes (*psbC* and *psbK*) were under positive selection. Maturase enzymes catalyze non-autocatalytic intron removal from premature RNAs, such as RNA transcripts for the *trnK*, *trnA*, *trnI*, *rps12*, *rpl2*, and *atpF* genes (Vogel et al., 1999). The *clpP* gene is a member of a gene family within the cp genome that encodes *clpP* proteases. In general, the *clpP* gene is essential for plant cells and the main function of its product is the degradation of polypeptides (Clarke, 1999; Shikanai et al., 2001; Kuroda and Maliga, 2003). We identified positively selected sites in the *clpP* gene in our study, which might have played key roles in the adaptive evolution of Dipsacales species. In addition, the *rbcL* gene plays an important role as a modulator of photosynthetic electron transport and it is essential for photosynthesis because it encodes the large subunit of RuBisCO (Allahverdiyeva et al., 2005). A previous study showed that *rbcL* is often under positive selection in land plants (Kapralov and Filatov, 2007). In particular, the *rbcL* gene evolved under strong positive selection after the C3–C4 photosynthetic transition (Piot et al., 2017). Similarly, selection analysis in *Haberlea rhodopensis* showed that 17 genes were under site-specific selection, and the *rbcL* gene harbored 13 sites under positive selection (Ivanova et al., 2017). We also found that the *ycf2* gene had 18 sites under positive selection, with 16 in *ycf1*, five in *rbcL*, three in *atpA*, and two in *clpP*, but only one site in each of the other 14 genes. These positively selected genes may have played key roles in the adaptation of species in the order Dipsacales to various environments.

### Phylogenetic Relationships

The phylogenetic trees based on five different datasets had similar topologies. In the order Dipsacales, two major lineages were clearly defined: Adoxaceae and Caprifoliaceae. *Viburnum* and *Sambucus* are the most closely related. In addition, the traditional Caprifolieae, Diervilleae, and Linnaeeae form a larger branch comprising the new family Caprifolieae. These results are similar to previous analyses based on morphological characters and various molecular data sets (Donoghue et al., 2001, 2003; Zhang et al., 2002; Winkworth et al., 2008). Zhang et al. (2002) suggested that Caprifoliaceae *s. l.* (excluding *Sambucus* and *Viburnum*) is a polyphyletic clade based on the sequence variations in *trnL* and *trnF* markers. In addition, other studies found that Morinaceae, Valerianaceae, and Dipsacaceae were derived from the polyphyletic Caprifoliaceae *s. l.*, which is divided into three separate lineages, i.e., Caprifoliaceae *s. str.*, Diervillaceae, and Linnaeaceae (Backlund and Pyck, 1998; Pyck et al., 1999). Our results showed that *W. florida* belongs to Diervilleae, *D. floribunda* and *K. amabilis* belong to Linnaeeae, and *T. pinnatifidum*, *L. tragophylla*, *L. stephanocarpa*, *L. japonica*, and *L. fragrantissima* var. *lancifolia* cluster in the Caprifoliaceae clade. Four *Lonicera* and one *Triosteum* species form a single clade in Caprifoliaceae with high bootstrap support, and *D. floribunda* and *K. amabilis* have a close relationship. These results agree well with previous evidence based on *trnL-F*, *ndhF*, and mitochondrial markers (Donoghue et al., 2001; Zhang et al., 2002; Winkworth et al., 2008). The similar topologies obtained based on various analyses, including these obtained in this study, indicate the clear resolution of the phylogenetic relationships in Dipsacales. In future research, it will be necessary to collect more species in order to verify the species relationships and interspecific divergence in Dipsacales.

### Molecular Dating

We estimated the divergence times of 14 Dipsacales species based on the protein-coding sequences in the complete cp genomes. The results showed that the diversification into Adoxaceae and Caprifoliaceae occurred about 81.14 mya at the Cretaceous/Tertiary boundary. These results are similar to the molecular divergence dates obtained in previous studies. For example, Bell and Donoghue (2005) used multiple methods to estimate the divergence dates of the major Dipsacales lineages, and suggested that the diversification of Adoxaceae and Caprifoliaceae mainly occurred in the Tertiary, and the major lineages mainly originated during the Eocene. In addition, in the Adoxaceae family, the split into *Viburnum* and *Sambucus* occurred in the Eocene. Within Caprifoliaceae, the splits to yield Diervilleae, Caprifolieae, and Linnaeeae also occurred in the Eocene period. Our results are similar to those obtained in previous studies. Smith et al. (2010) estimated the divergence date for *Lonicera* and suggested that an ancestor of this genus had a widespread distribution across the Northern Hemisphere about 7–17 mya. We found that the spilt to yield *L. tragophylla* (mean age = 19 mya) occurred earlier than that for other *Lonicera* species. To the best of our knowledge, the present study is the first to use all of the protein-coding sequences in cp genomes to estimate the divergence dates of Dipsacales, although the results could be improved by larger phylogenetic analyses.

## Author Contributions

Z-HL conceived and designed the study. W-BF and JY performed the experiments. Z-HL, W-BF, YW, and KS contributed materials and analysis tools. W-BF and Z-HL wrote the paper. Z-HL and W-BF revised the paper. All authors approved the final manuscript.

## Conflict of Interest Statement

The authors declare that the research was conducted in the absence of any commercial or financial relationships that could be construed as a potential conflict of interest.

## Supplementary Material

FIGURE S1 Sequence alignment of chloroplast genomes from 14 Dipsacales species. Sequences of chloroplast genomes were aligned and compared using the mVISTA program. The vertical scale indicates the percentage identity, ranging from 50% to 100%.

FIGURE S2 Phylogenetic relationships among the 16 species inferred by maximum likelihood and Bayesian analyses based on five datasets. **(A)** Complete chloroplast genome; **(B)** Coding region; **(C)** LSC region; **(D)** SSC region; and **(E)** IR region. The numbers to the right of the slashes on the braches show the bootstrap values obtained by maximum likelihood analyses, and those to the left show the posterior probabilities according to Bayesian inference.

TABLE S1 Sampling and assembly information for the 16 species.

TABLE S2 Primers for low coverage regions in seven Dipsacales species.

TABLE S3 List of tandem repeats in the chloroplast genomes of 14 Dipsacales species.

TABLE S4 List of dispersed repeats and palindromic repeats in the chloroplast genomes of 14 Dipsacales species.

TABLE S5 Percentages of variable characters in coding and non-coding regions within 14 Dipsacales species.

TABLE S6 Maximum likelihood parameter estimates for 75 genes in Dipsacales.

TABLE S7 Maximum likelihood parameter estimates for 19 genes with positive selection sites.

TABLE S8 List of genes present in the chloroplast genomes of six Adoxaceae species and eight Caprifoliaceae species: **(a)** two gene copies in Caprifoliaceae; **(b)** two gene copies in Adoxaceae; **(c)** pseudogene in the chloroplast genome of Caprifoliaceae; and **(d)** only present in Caprifoliaceae.

TABLE S9 Likelihood ratio test (LRT) of the variable ω ratio under different models.
