# Supplementary figures and images for: Comparative Chloroplast Genomics of Dipsacales Species: Insights Into Sequence Variation, Adaptive Evolution, and Phylogenetic Relationships
Source: Front Plant Sci. 2018 May 23;9:689. doi: 10.3389/fpls.2018.00689 (PMC5974163; doi:10.3389/fpls.2018.00689)

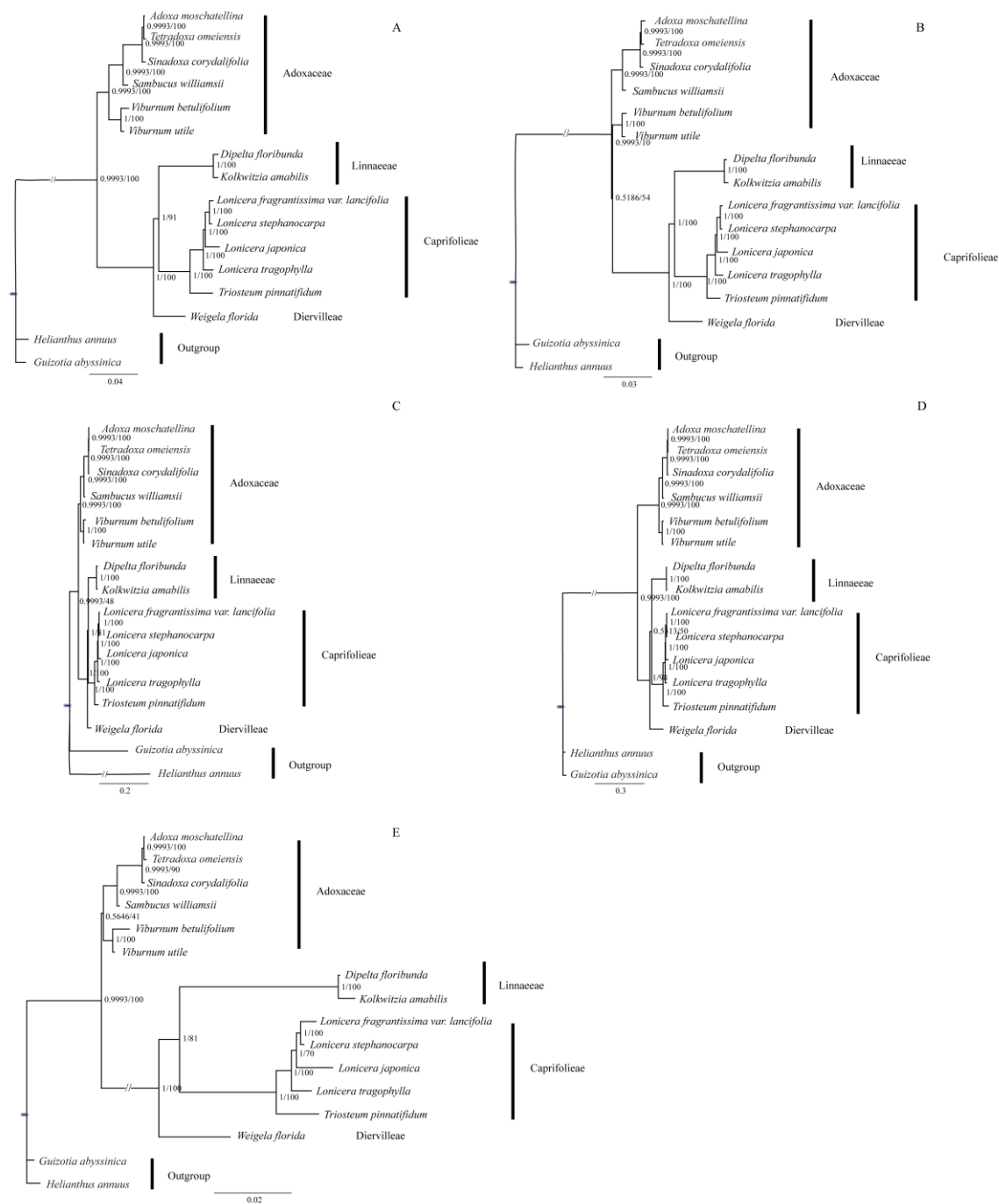

**Fig. S2**

Supplement: FIGURE S1 — Sequence alignment of chloroplast genomes from 14 Dipsacales species. Sequences of chloroplast genomes were aligned and compared using the mVISTA program. The vertical scale indicates the percentage identity, ranging from 50% to 100%. [file Image_1.PDF]
