# Supplementary material for: Comparative Chloroplast Genomics of Dipsacales Species: Insights Into Sequence Variation, Adaptive Evolution, and Phylogenetic Relationships
Source: Front Plant Sci. 2018 May 23;9:689. doi: 10.3389/fpls.2018.00689 (PMC5974163; doi:10.3389/fpls.2018.00689)
Supplement: TABLE S1 — Sampling and assembly information for the 16 species. [file Table_1.DOCX]

**Table S1 Sampling and assembly information for the 16 species.**

| No. | Species | Locality | PE150/PE125 | Clean reads (M) | Clean reads (Q30) | Assembly reads | Mean length of reads | Mean coverage | Accession number in GenBank |
| --- | --- | --- | --- | --- | --- | --- | --- | --- | --- |
| 1 | *Viburnum betulifolium* | Xi’an, Shaanxi, China | 158 | 18.524 | 95.9% | 125,556 | 151.3 | 119.1 | MG738665 |
| 2 | *Lonicera fragrantissima* var*. lancifolia* | An’kang, Shaanxi, China | 120 | 23.122 | 96.6% | 815,541 | 148.8 | 782.3 | MG738669 |
| 3 | *Lonicera stephanocarpa* | Xi’an, Shaanxi, China | 132 | 23.659 | 96.7% | 983,391 | 148.7 | 944.6 | MG738668 |
| 4 | *Lonicera tragophylla* | Xi’an, Shaanxi, China | 121 | 22.047 | 96.4% | 746,663 | 147.9 | 702.9 | MG738667 |
| 5 | *Triosteum pinnatifidum* | Baoji, Shaanxi China | 158 | 20.096 | 95.6% | 131,051 | 151.2 | 125.0 | MG738666 |
| 6 | *Weigela florida* | Xi’an, Shaanxi, China | 158 | 13.104 | 96.7% | 752,480 | 148.2 | 713.5 | MG738664 |
| 7 | *Dipelta floribunda* | Xi’an, Shaanxi, China | 158 | 22.967 | 96.8% | 1,265,346 | 150.0 | 1,198.4 | MG738670 |
| 8 | *Viburnum utile* |  |  |  |  |  |  |  | NC_032296 |
| 9 | *Sambucus williamsii* |  |  |  |  |  |  |  | NC_033878 |
| 10 | *Sinadoxa corydalifolia* |  |  |  |  |  |  |  | NC_029874 |
| 11 | *Lonicera japonica* |  |  |  |  |  |  |  | KX258652 |
| 12 | *Kolkwitzia amabilis* |  |  |  |  |  |  |  | NC_010442 |
| 13 | *Adoxa moschatellina* |  |  |  |  |  |  |  | NC_007898 |
| 14 | *Trachelium caeruleum* |  |  |  |  |  |  |  | NC_032040 |
| 15 | *Helianthus annuus* |  |  |  |  |  |  |  | NC_007977 |
| 16 | *Guizotia abyssinica* |  |  |  |  |  |  |  | NC_010601 |
