# Supplementary material for: Comparative Chloroplast Genomics of Dipsacales Species: Insights Into Sequence Variation, Adaptive Evolution, and Phylogenetic Relationships
Source: Front Plant Sci. 2018 May 23;9:689. doi: 10.3389/fpls.2018.00689 (PMC5974163; doi:10.3389/fpls.2018.00689)
Supplement: TABLE S2 — Primers for low coverage regions in seven Dipsacales species. [file Table_2.DOCX]

| **Table S2 Primers for low coverage regions in seven Dipsacales species.** | | | | |
| --- | --- | --- | --- | --- |
| **No.** | **Region amplified** | **Forward sequence (5' to 3')** | **Reverse sequence (5' to 3')** | **Ta (℃)** |
| *L. fragrantissima* var*. lancifolia* | *trnI-CAU-trnH-GUG* | TTAAGCATCCATGGCTGAAT | GATCAAGGCAGTGGATTGT | 52 |
| *L. tragophylla* | *trnI-CAU-trnH-GUG* | TAAAGCGCCCAACTCATAAT | GATCAAGGCAGTGGATTGT | 52 |
| *L. stephanocarpa* | *trnI-CAU-trnH-GUG* | GAATTCGTAGGTTCAATTCC | GATCAAGGCAGTGGATTGT | 52 |
| *V. betulifolium* | *trnI-CAU-trnH-GUG* | GGTAAGCGTCCTGTAGTAAG | ACAATCCACTGCCTTGATC | 54 |
| *D. floribunda* | *trnI-CAU-trnH-GUG* | GAATTCGTAGGTTCAATTCC | TCCTCAAGTCGAATCCTGAT | 52 |
| *W. florida* | *trnI-CAU-trnH-GUG* | GAATTCGTAGGTTCAATTCC | CAAGGCAGTGGATTGTGAAT | 52 |
| *L. tragophylla* | *accD gene* | GAAGGACCCTAGCTATGGA | TGTATAACACTCATCGCACA | 53 |
| *L. stephanocarpa* | *accD gene* | AAGTTAAGGAGGAGATTCGC | TGAAATCCACATCCTTCACA | 52 |
| *V. betulifolium* | *trnC-GCA-petN* | GTGTCGCCTGATCAACAAA | AGCGAGACTTACTATATCCA | 52 |
| *D. floribunda* | *rbcL-accD* | ACACGACATAGCAGAAATGA | TCGCATAGAACCCACAAATG | 52 |
| *T. pinnatifidum* | *rbcL-accD* | TGTTTAACACGACATAGCA | CCCTTGCAAATCTGATTTTCA | 50 |
