# Supplementary material for: Comparative Chloroplast Genomics of Dipsacales Species: Insights Into Sequence Variation, Adaptive Evolution, and Phylogenetic Relationships
Source: Front Plant Sci. 2018 May 23;9:689. doi: 10.3389/fpls.2018.00689 (PMC5974163; doi:10.3389/fpls.2018.00689)
Supplement: TABLE S8 — List of genes present in the chloroplast genomes of six Adoxaceae species and eight Caprifoliaceae species: (a) two gene copies in Caprifoliaceae; (b) two gene copies in Adoxaceae; (c) pseudogene in the chloroplast genome of Caprifoliaceae; and (d) only present in Caprifoliaceae. [file Table_8.DOCX]

**Table S8 List of genes present in the chloroplast genomes of six Adoxaceae species and eight Caprifoliaceae species.**

| Gene group | Gene name |  |  |  |  |
| --- | --- | --- | --- | --- | --- |
| Ribosomal RNA genes | *rrn16* (ab) | *rrn23* (ab) | *rrn4.5* (ab) | *rrn5* (ab) |  |
| Transfer RNA genes | *trnI-CAU* (ab) | *trnI-GAU* (ab) | *trnL-UAA* | *trnL-CAA* (ab) | *trnL-UAG* |
|  | *trnR-UCU* | *trnR-ACG* (ab) | *trnA-UGC* (ab) | *trnW-CCA* | *·* |
|  | *trnV-UAC* | *trnV-GAC*(ab) | *trnF-GAA* | *trnT-UGU* | *trnT-GGU* |
|  | *trnP-UGG* | *trnfM-CAU* | *trnP-GGG* | *trnG-GCC* | *trnS-GGA* |
|  | *trnS-UGA* | *trnS-GCU* | *trnD-GUC* | *trnC-GCA* | *trnN-GUU* (ab) |
|  | *trnE-UUC* | *trnY-GUA* | *trnQ-UUG* | *trnK-UUU* | *trnH-GUG* |
|  |  |  |  |  |  |
| Small Subunit of ribosome | *rps2* | *rps3* | *rps4* | *rps7* (ab) | *rps8* |
|  | *rps11* | *rps12* (ab) | *rps14* | *rps15* | *rps16* |
|  | *rps18* | *rps19* |  |  |  |
| Large Subunit of ribosome | *rp12* (b) | *rp114* | *rp116* | *rp120* | *rp122* |
|  | *rp123* (b) | *rp132* | *rp133* | *rp136* |  |
| DNA-dependent RNA polymerase | *rpoA* | *rpoB* | *rpoC1* | *rpoC2* |  |
| Translational initiation factor | *infA* |  |  |  |  |
| Subunits of photosystem I | *psaA* | *psaB* | *psaC* | *psaI* | *psaJ* |
|  | *ycf3* | *ycf4* |  |  |  |
| Subunits of photosystem II | *psbB* | *psbC* | *psbD* | *psbE* | *psbF* |
|  |  |  |  |  |  |
|  | *psbH* | *psbI* | *psbJ* | *psbL* | *psbM* |
|  | *psbN* | *psbT* |  |  |  |
| NADH oxidoreductase | *ndhA* | *ndhB* (ab) | *ndhC* | *ndhD* | *ndhE* |
|  | *ndhG* | *ndhI* | *ndhJ* | *ndhK* | *ndhF* (d) |
| Subunits of cytochrome | *petA* | *petB* | *petD* | *petG* | *petL* |
|  | *petN* |  |  |  |  |
| Subunits of ATP synthase | *atpA* | *atpB* | *atpE* | *atpF* | *atpH* |
|  | *atpI* |  |  |  |  |
| Large subunit of Rubisco | *rbcL* |  |  |  |  |
| Maturase | *matk* |  |  |  |  |
| Envelope membrane protein | *cemA* |  |  |  |  |
| Subunit of acetyl-CoA | *accD* (c) |  |  |  |  |
| C-type cytochrome synthesis gene | *ccsA* |  |  |  |  |

Note: (a) two gene copies in Caprifoliaceae; (b) two gene copies in Adoxaceae; (c) pseudogene in the chloroplast genome of Caprifoliaceae; and (d) only present in Caprifoliaceae.
