# Supplementary material for: Comparative Chloroplast Genomics of Dipsacales Species: Insights Into Sequence Variation, Adaptive Evolution, and Phylogenetic Relationships
Source: Front Plant Sci. 2018 May 23;9:689. doi: 10.3389/fpls.2018.00689 (PMC5974163; doi:10.3389/fpls.2018.00689)
Supplement: TABLE S9 — Likelihood ratio test (LRT) of the variable ω ratio under different models. [file Table_9.DOCX]

**Table S9 Likelihood ratio test (LRT) of the variable ω ratio under different models.**

| gene | comparisons | 2Δl | df | p |
| --- | --- | --- | --- | --- |
| *atpA* | M1 vs M2 | 45.63542 | 2 | 1.23E-10 |
|  | M7 vs M8 | 46.82886 | 2 | 6.78E-11 |
| *atpB* | M1 vs M2 | 48.05579 | 2 | 3.67E-11 |
|  | M7 vs M8 | 48.15499 | 2 | 3.49E-11 |
| *atpI* | M1 vs M2 | 54.46772 | 2 | 1.49E-12 |
|  | M7 vs M8 | 55.66919 | 2 | 8.16E-13 |
| *clpP* | M1 vs M2 | 20.45975 | 2 | 3.61E-05 |
|  | M7 vs M8 | 22.73638 | 2 | 1.16E-05 |
| *infA* | M1 vs M2 | 9.583858 | 2 | 0.008296 |
|  | M7 vs M8 | 11.64183 | 2 | 0.002965 |
| *matK* | M1 vs M2 | 14.54355 | 2 | 0.000695 |
|  | M7 vs M8 | 15.20126 | 2 | 0.0005 |
| *psaA* | M1 vs M2 | 38.7283 | 2 | 3.89E-09 |
|  | M7 vs M8 | 41.00102 | 2 | 1.25E-09 |
| *psaJ* | M1 vs M2 | 46.91317 | 2 | 6.50E-11 |
|  | M7 vs M8 | 46.91659 | 2 | 6.49E-11 |
| *psbC* | M1 vs M2 | 39.54918 | 2 | 2.58E-09 |
|  | M7 vs M8 | 41.49856 | 2 | 9.74E-10 |
| *psbK* | M1 vs M2 | 53.26234 | 2 | 2.72E-12 |
|  | M7 vs M8 | 53.6426 | 2 | 2.25E-12 |
| *rbcL* | M1 vs M2 | 61.49584 | 2 | 4.43E-14 |
|  | M7 vs M8 | 61.21313 | 2 | 5.10E-14 |
| *rpl22* | M1 vs M2 | 10.721 | 2 | 0.004699 |
|  | M7 vs M8 | 10.88983 | 2 | 0.004318 |
| *rpl32* | M1 vs M2 | 30.53565 | 2 | 2.34E-07 |
|  | M7 vs M8 | 30.27132 | 2 | 2.67E-07 |
| *rps3* | M1 vs M2 | 20.15848 | 2 | 4.19E-05 |
|  | M7 vs M8 | 22.23722 | 2 | 1.48E-05 |
| *rps7* | M1 vs M2 | 10.07167 | 2 | 0.006501 |
|  | M7 vs M8 | 12.01409 | 2 | 0.002461 |
| *rps14* | M1 vs M2 | 53.40531 | 2 | 2.53E-12 |
|  | M7 vs M8 | 53.70724 | 2 | 2.18E-12 |
| *rps15* | M1 vs M2 | 22.50177 | 2 | 1.30E-05 |
|  | M7 vs M8 | 23.6641 | 2 | 7.27E-06 |
| *ycf1* | M1 vs M2 | 142.9106 | 2 | 9.28E-32 |
|  | M7 vs M8 | 146.5344 | 2 | 1.52E-32 |
| *ycf2* | M1 vs M2 | 177.1966 | 2 | 3.33E-39 |
|  | M7 vs M8 | 176.631 | 2 | 4.42E-39 |
